# 

**Published:** 1883-07

**Authors:** 


### CONTENTS.

BIOGRAPHICAL.

Wm. H. Goddard, D.D.S., Louisville, Kentucky .......... 307

COMMUNICATIONS.

Empiricism .......... 315
Helps in Study .......... 321
What Should be Done Further to Protect the Public from the Increasing Empiricism Practiced in our Large Cities .......... 326
Description of a Novel Tooth-Crown .......... 331
Germs and Acids in Decay of the Teeth .......... 335

SELECTIONS.

Anæsthetics—Use and Results .......... 338
Eyesight of School Children .......... 344

PROCEEDINGS.

Kentucky State Dental Association .......... 346
In Memory .......... 348

EDITORIAL.

Meeting of the Board of Examiners .......... 349
Pennsylvania State Dental Society .......... 349
Another Law Regulating Dental Practice .......... 349
Dressing Disks .......... 353
Educational .......... 354
American Dental Association .......... 354
Ho! for Niagara .......... 356

